# Inner-ear delivery of AAV2-retro robustly labels lateral olivocochlear efferents but results in off-target transfection in the contralateral cochlea and the brain

**DOI:** 10.3389/fgeed.2026.1946992

**Published:** 2026-10-19

**Authors:** Mahmoud Khalil, Mikayla Bisignani, Karl Kandler

**Affiliations:** 1 Department of Neurobiology, University of Pittsburgh School of Medicine, Pittsburgh, PA, United States; 2 University of Pittsburgh School of Medicine, Pittsburgh, PA, United States; 3 Department of Bioengineering, University of Pittsburgh, Pittsburgh, PA, United States

**Keywords:** AAV serotype, AAV2-retro, adeno-associated viruses, inner ear gene delivery, inner ear injection, lateral olivocochlear pathway, LOC

## Abstract

Gene delivery via adeno-associated viruses (AAVs) is a valuable tool for understanding the organization and function of auditory neuronal circuitry. In this study, we explored the use of AAVs injected into the inner ear of mice to transfect the lateral olivocochlear (LOC) pathway, a poorly understood ipsilateral component of the auditory efferent feedback system. AAV serotypes exhibit a wide range of properties, including cell type selectivity and the capacity for retrograde transport, but none of the AAV serotypes used for inner ear delivery have been reported to successfully transfect the LOC pathway. Here, we show that unilateral inner ear delivery of AAV2-retro robustly labeled the LOC pathway. However, the ability of AAV2-retro to diffuse via the cochlear aqueduct from the perilymph into the cerebrospinal fluid resulted in off-target transfection in the brain and the labeling of LOC projections in the contralateral cochlea. The extent of contralateral transfection of LOC neurons depended on the age of the animal and the amount of virus delivered to the inner ear. These findings highlight the importance of considering the dosage and properties of the AAV serotype when interpreting experimental results.

## 1 Introduction

Adeno-associated viruses (AAVs) are an indispensable tool for neuroscience research with applications ranging from circuit tracing (Luchicchi et al., 2021; Saleeba et al., 2019; Weinholtz and Castle, 2021) and circuit manipulation (Watakabe et al., 2017) to gene therapy (Deverman et al., 2018). In the field of auditory neuroscience, inner ear delivery of AAVs has been used to develop potential therapeutic approaches to rescuing hearing loss (Hao and Li, 2019). AAV-mediated gene therapy has been successfully applied to restore hearing in congenitally deaf mice by delivering functional genes to hair cells (Pan et al., 2017; Akil et al., 2012) or through the delivery of miRNA to rescue autosomal dominant hearing loss (Shibata et al., 2016).

Different serotypes of AAV vary in their tropism for different cell types (Aschauer et al., 2013; Sun et al., 2019; Witteveen and Balmer, 2024), their capacity for anterograde and retrograde transport (Rothermel et al., 2013; Wang and Zhang, 2021), their efficacy of viral transduction (Aschauer et al., 2013), and their local diffusion from the injection site (Taymans et al., 2007; Witteveen and Balmer, 2024). Recent studies have identified AAV variants that are suited for AAV-mediated gene delivery in the inner ear by developing variants that exhibit lower toxicity and higher efficacy of transfecting hair cells and supporting cells in the injected ear (Ivanchenko et al., 2021; Landegger et al., 2017; Isgrig et al., 2019).

In this study, we tested the suitability of inner ear delivery of AAVs to retrogradely transduce the efferent lateral olivocochlear (LOC) pathway. The LOC pathway and the medial olivocochlear (MOC) pathway are the two components of efferent innervation in the mammalian cochlea (Warr and Guinan, 1979; Guinan et al., 1983; White and Warr, 1983; Sánchez-González et al., 2003). LOC neurons can be subdivided into dopaminergic shell LOC neurons which are located around the boundary of the lateral superior olive (LSO) and cholinergic core LOC neurons which are located inside the LSO nucleus (Darrow et al., 2006b; Warr et al., 1997; Brown, 1987). Core LOC neurons represent 80%–95% of all LOC neurons depending on species (Darrow et al., 2006b; Horváth et al., 2000; Vetter and Mugnaini, 1992). Both core and shell LOC neurons innervate the afferent synapses at the inner hair cell region (Wilson et al., 1991; Liberman, 1980), with core LOC projections forming dense arborizations along tonotopically restricted regions and shell LOC projections bifurcating along the cochlear axis to cover a wide tonotopic region with en passant swellings instead of dense terminal arbors (Warr et al., 1997; Brown, 1987). Additionally, the efferent projections of core LOC neurons are almost exclusively ipsilateral, while 5%–20% of shell LOC neurons also project to the contralateral cochlea (Horváth et al., 2000; Vetter and Mugnaini, 1992).

Previous studies suggest that the LOC pathway plays a role in protecting against both age- and noise-induced synaptopathy (Liberman et al., 2014; Darrow et al., 2007), in learning (Irving et al., 2011), and in balancing interaural inputs (Darrow et al., 2006a). In humans, the function of LOC efferents was speculated to be involved in the balancing of binaural input in tinnitus patients (Cho et al., 2022) and in auditory symptoms associated with Parkinson’s disease (Pisani et al., 2015). However, a better understanding of LOC pathway function and its involvement in pathology has been hampered by the lack of a biological readout specific to LOC pathway activity and the difficulty of selectively targeting LOC neurons without impacting other components of the auditory system. Anatomical lesions of either the LSO nucleus, where LOC neurons reside (Darrow et al., 2006b; Liberman et al., 2014), or the olivocochlear bundle that contains LOC axons (Irving et al., 2011) are not specific to LOC neurons. Additionally, the shared cholinergic identity between most MOC and LOC neurons limits avenues for pharmacological manipulations. Targeting the LOC pathway through inner ear delivery of AAV could open up additional approaches to overcome these hurdles. However, AAV serotypes commonly used for gene delivery to the inner ear have not been reported to label the LOC pathway (Kilpatrick et al., 2011; Blanc et al., 2022; Marcovich et al., 2022). Identifying a suitable AAV serotype for transducing the LOC pathway would, therefore, be a promising first step. A major challenge in identifying a suitable serotype, however, is that the capacity for retrograde transport and selectivity for neuronal subtypes varies widely among AAV serotypes (Rothermel et al., 2013; Wang and Zhang, 2021).

AAV2-retro is an AAV variant capable of retrograde neuronal transduction that was created by modifying the capsid of the naturally occurring AAV2 serotype (Tervo et al., 2016). AAV2-retro exhibits variable cellular tropism, however, as it shows diminished retrograde transduction capacity for some neuron types (Han et al., 2023; Skorput et al., 2022; Wang et al., 2018) while failing to transduce others (Bortoloci and Motta, 2022). When Wang et al. (2021) delivered AAV2-retro to the inner ear, they observed transduction in MOC and shell LOC neurons but did not report evidence of transduction in core LOC neurons (Wang et al., 2021). While this observation could be reflective of AAV2-retro’s tropism, successful AAV transduction could also be impacted by other factors like the specific promoter used to drive gene expression (Kügler et al., 2003; Bohlen et al., 2020) and the titer used (Lu et al., 2024). Since AAV2-retro is one of the most readily available AAV serotypes capable of robust retrograde transduction, we set out to investigate whether it can retrogradely transduce core LOC neurons. Our results demonstrate that inner ear delivery of AAV2-retro results in robust retrograde transduction of core LOC neurons in both neonatal and juvenile mice. Additionally, we report that unilateral delivery of AAV2-retro results in bilateral transduction of core LOC neurons, which most likely occurred through the diffusion of viral particles via the cochlear aqueduct into the cerebrospinal fluid and the contralateral ear. The extent of AAV2-retro transduction in the contralateral cochlea was volume- and age-dependent. Taken together, our results demonstrate that AAV2-retro is a viable method of retrogradely transducing the LOC pathway but that its proclivity to diffuse throughout the central nervous system should be taken into account when designing experiments and interpreting results.

## 2 Materials and methods

### 2.1 Animals

All experiments were performed on C57BL/6J mice of both sexes. All experimental procedures were in accordance with NIH guidelines and were approved by the institutional animal care and use committee at the University of Pittsburgh.

### 2.2 Inner ear delivery of AAV and Fast Blue

Surgeries for inner ear delivery of AAV were performed on animals aged postnatal day (P) 1-3 and P21-28. In neonates, r-AAV2-retro-hSyn-mCherry (AAV2-retro; Addgene 114472-AAVrg; titer 2e+13 GC/mL) or Fast Blue (Polysciences, Inc catalogue number: 17740) was delivered to the inner ear through injections into the posterior semicircular canal (Isgrig and Chien, 2018). P1-3 mouse pups were anesthetized using hypothermia until the cessation of movement and a lack of response to tail pinch. Hypothermic anesthesia was maintained for the duration of the surgery by placing the pup on an ice pack wrapped with a sterile absorbent pad. An incision was made behind the ear flap to expose the sternocleidomastoid muscles, which were dissected to expose the cartilaginous structure of the posterior semicircular canal. AAV was injected into the semicircular canal with a pulled glass micropipette attached to a stereotaxic mount. Injections were made at a rate of 1 nL/s (Nanoject III) or with pulses of 2 nL at 23 nL/s every 3 seconds (Nanoject II). Total volume injected in neonates was 360-400 nL for Figure 1, and 200 nL or 50 nL as indicated in later figures. After completing the injection, the pipette was removed, and the incision was sutured with an 8/0 monofilament nylon suture (Fine Science Tools). Pups were then placed on a warming pad and returned to their mother once they recovered from anesthesia and gained mobility.

**FIGURE 1:**
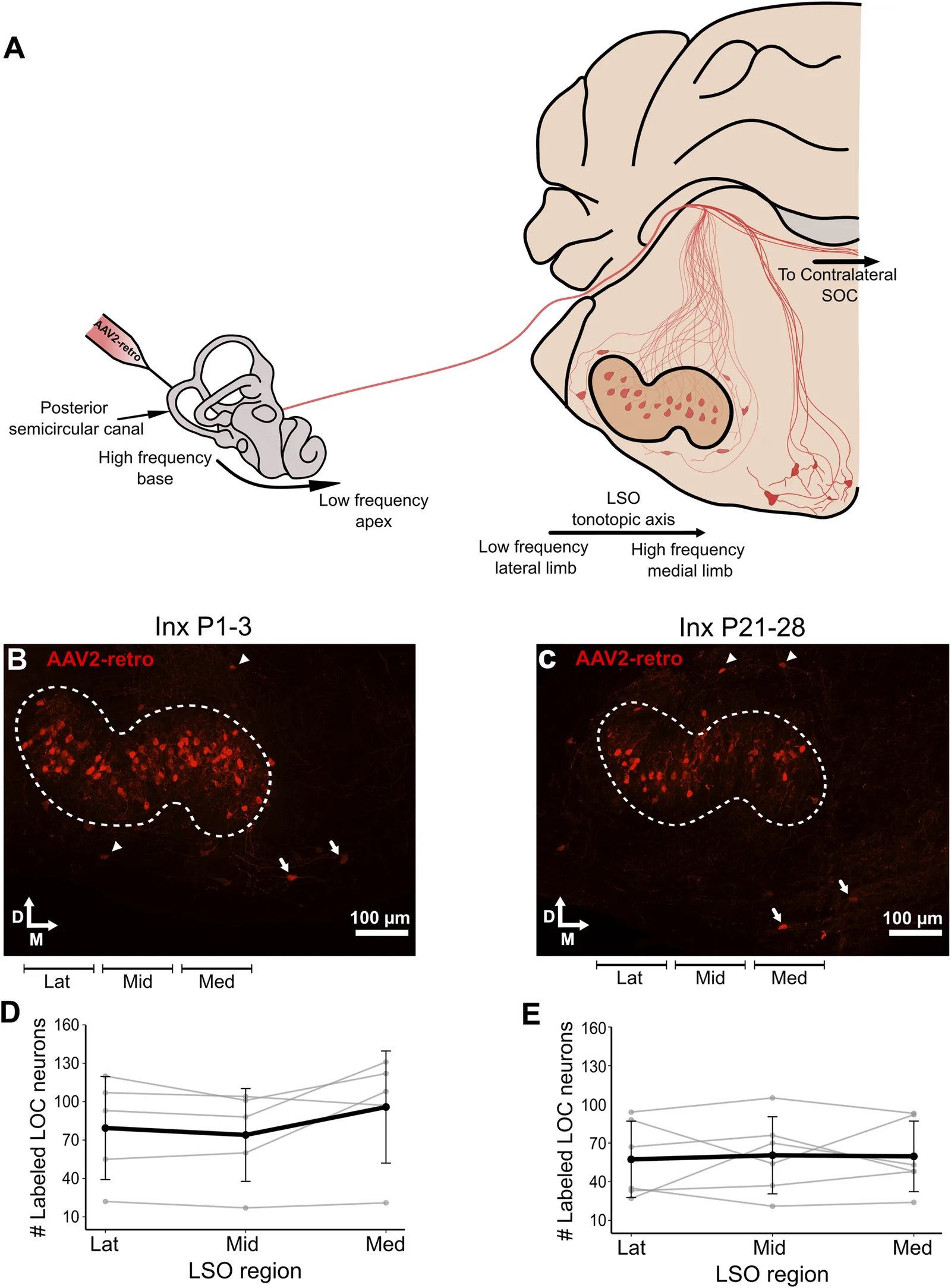
Inner ear delivery of AAV2-retro robustly labels LOC neurons along the tonotopic axis of the LSO. **(A)** Overview of the lateral olivocochlear pathway and experimental approach. **(B)** Retrogradely labeled core LOC neurons in the ipsilateral LSO following neonatal inner ear delivery of AAV2-retro. D = dorsal. M = medial. Arrowheads: Shell LOC neurons. Arrows: MOC neurons, Lat: Lateral. Mid: Middle. Med: Medial. Inx: Injection **(C)** Retrogradely labeled core LOC neurons in the ipsilateral LSO following inner ear delivery of AAV2-retro in P21–28 animals. **(D)** Quantification of retrogradely labeled LOC neurons in the lateral, middle, and medial regions of the ipsilateral LSO following P1-3 inner ear delivery of AAV2-retro (N = 5). Thin line–individual cases, thick line average. Lateral region: 79 ± 40, middle region: 74 ± 36, medial region: 95 ± 43. one-way repeated measures ANOVA, F = 2.76 P = 0.12. **(E)** Quantification of retrogradely labeled LOC neurons in the lateral, middle, and medial regions of the ipsilateral LSO following P21-28 inner ear delivery of AAV2-retro (N = 6). Lateral region: 57 ± 29, middle region: 59 ± 27, medial region: 60 ± 29 one-way repeated measures ANOVA, F = 0.064 P = 0.937.

P21 and older mice were anesthetized by intraperitoneal injection of 100 mg/kg ketamine and 10 mg/kg xylazine. AAV injections into the posterior semicircular canal were similar to Suzuki et al. (2017). A postauricular incision was made and a small incision through the underlying lipid and connective tissue exposed the sternocleidomastoid muscles, which were dissected in order to expose the posterior semicircular canal. A small hole was hand-drilled in the semicircular canal using a 27-gauge hypodermic needle. Perilymph was allowed to drain for 5–10 min and was soaked up using a sterile Gelfoam sponge (Pfizer Injectables, manufactured and distributed by Pharmacia & Upjohn). A 0.0030’’/0.0040″ ID/OD Polyimide catheter tube (manufactured by Teleflex Medical OEM) attached to a glass pipette with UV curable epoxy was then inserted into the posterior semicircular canal. The opening through which the polyimide catheter was inserted was sealed using fragments of muscle tissue and cyanoacrylate glue (3M Vetbond Tissue Adhesive). Injections were performed at a rate of 1 nL/s (Nanoject III) or with pulses of 23 nL at 23 nL/s every 20 s (Nanoject II). Total injection volume in juvenile mice was 1.2 μL. After withdrawal of the catheter, the hole in the semicircular canal was sealed with muscle fragments and cyanoacrylate glue (3M Vetbond Tissue Adhesive). The surgery site was sutured with a 5-0, FS-2, 19 mm reverse cutting, Perma-Hand Silk suture (Ethicon). Lidocaine cream was applied over the sutured incision to remedy pain, and for 3 days, animals received daily subcutaneous injections of 10 mg/kg ketoprofen.

### 2.3 Immunocytochemistry for AAV and Fast Blue tracing experiments

21–22 days after AAV injections and 14 days after Fast Blue injections, animals were anesthetized with 100 mg/kg ketamine and 10 mg/kg xylazine and perfused transcardially with phosphate buffered saline (PBS) followed by 4% paraformaldehyde in PBS (4% PFA). The brains and temporal bones were then dissected and post-fixed in 4% PFA in PBS solution overnight at 4 °C followed by submersion in 30% sucrose in PBS for cryopreservation for 48 h at 4 °C. Coronal sections (50 µm thick) of the superior olivary complex (SOC) were prepared using a freezing microtome (Microm HM 430). Temporal bones underwent cochlear whole-mount dissections using fine forceps (Haque et al., 2015).

Brain sections from animals injected with Fast Blue were washed in PBS (2x for 5 min) and were mounted on Superfrost Plus microscope slides (Fisherbrand) using Fluoromount-G (Southern Biotech) and coverslipped.

Brain sections and cochlea tissue from AAV tracing animals were washed in PBS (2x for 5 min) and then incubated in blocking solution (1% BSA, 5% donkey serum, 0.1% triton-x) overnight at 4 °C. Both primary and secondary antibodies were prepared in blocking solution. The following antibodies were used: 1:500 Scigen Goat anti-tdTomato (Lot#: 0081030221) and 1:1000 Invitrogen Alexa Fluor 568 donkey anti-goat (Ref#: A11057, Lot#:2304269).

Following blocking, brain sections and cochlea tissue were incubated in primary antibody solution overnight at 4 °C on a stirrer plate, washed in PBS (5x for 10 min), and incubated in secondary antibody solution overnight at 4 °C on a stirrer plate. Sections were washed in PBS (5x for 10 min), mounted on Superfrost Plus microscope slides (Fisherbrand) using Fluoromount-G (Southern Biotech) and coverslipped.

### 2.4 DiI tracing

P2-3 mouse pups were anesthetized with 100 mg/kg ketamine and 10 mg/kg xylazine and transcardially perfused with PBS followed by 4% paraformaldehyde in PBS (4% PFA). An incision was made ventrocaudal to the ear flap, and the underlying tissue was dissected in order to expose the auditory bulla. Individual cotton threads separated from Curity gauze sponges (Covidien) infused with DiI were then inserted through the auditory bulla and into the cochlea using fine syringes. Threads were infused with DiI by soaking them with 1,1′-Dioctadecyl-3,3,3′,3′-tetramethyl-indocarbocyanine perchlorate (DiI, Sigma-Aldrich, Lot# MKCDO794) dissolved in 70% ethanol and subsequent air drying. Tissues were then stored in a 4% PFA/PBS solution in the dark at room temperature and after 14–15 weeks, the brains were extracted and sliced coronally (50 µm) in PBS using a Leica VT1000S Vibratome. Sections containing the SOC were mounted on Superfrost Plus microscope slides (Fisherbrand) using Fluoromount-G (Southern Biotech) and coverslipped.

### 2.5 Microscopy and quantification of cells

Digital micrographs of the SOC and cochlea were obtained using a fluorescent microscope (Axio Imager, Zeiss) or a confocal microscope (Nikon A1). Sections were first visualized through the eyepiece with epifluorescence illumination. The different background autofluorescence properties of brain tissue structures allowed us to delineate the anatomical landmarks and nuclei of the SOC. The LSO nucleus was identified by its S-shaped structure, medial to the facial nerve root and dorsolateral to the medial nucleus of the trapezoid body. The LSO typically spanned 8–9 brain sections, all of which were imaged and quantified. Labeled cells were counted manually using the “multi-point” tool on ImageJ version 2.9.0/1.54f. The identity of retrogradely labeled olivocochlear neurons was determined based on anatomical location. Core LOC neurons were defined as retrogradely labeled neurons located within the LSO. The lateral, middle, and medial regions of the LSO were determined by rotating the LSO in each section to a horizontal position and dividing it along the horizontal axis into three equal-length regions. Shell LOC neurons were defined as bi- and multi-polar cells located around the periphery of the LSO nucleus. MOC neurons were defined as retrogradely labeled cells located in the ventral nucleus of the trapezoid body, medioventral to the LSO.

### 2.6 Statistical analysis

RStudio was used to conduct all the statistical analysis and generate plots. Plots were created using the tidyverse and ggplot2 packages. Data was tested for normality by the Shapiro–Wilk normality test using the base R stats package. Statistical comparisons were performed using either Student’s t-tests or ANOVA (base R stats package). Tukey’s HSD post-hoc comparisons were performed if the ANOVA results were significant by using the emmeans package. The Holm–Bonferroni correction for multiple comparisons was performed to correct the P-values reported for Figures 2D–F,J–L using the base R stats package.

**FIGURE 2:**
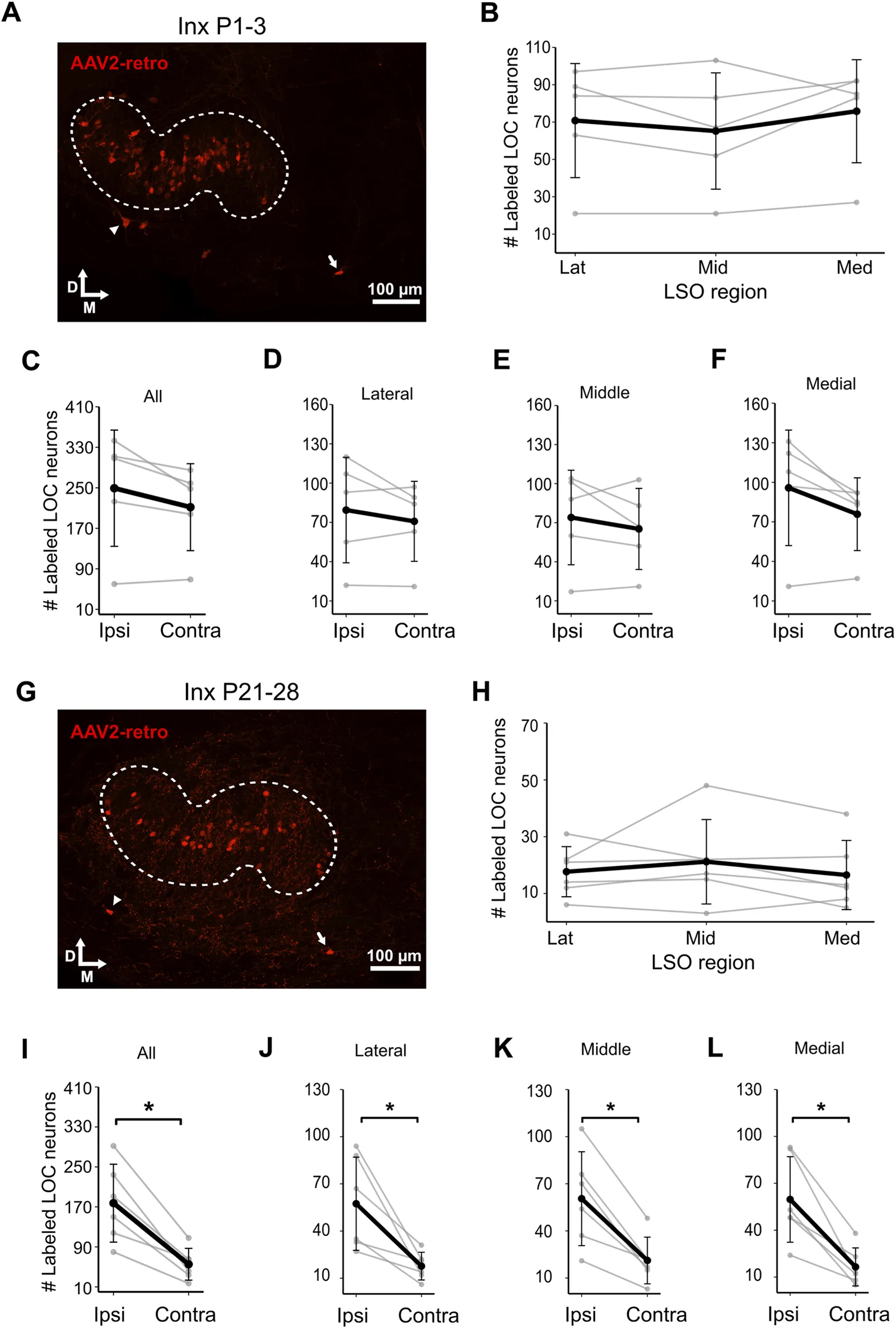
Unilateral inner ear injection of AAV2-retro labels LOC neurons in the contralateral LSO. **(A)** Retrogradely labeled core LOC neurons in the contralateral LSO in neonatally injected mice. D = dorsal. M = medial. Arrowheads: Shell LOC neurons. Arrows: MOC neurons, Lat: Lateral. Mid: Middle. Med: Medial. Inx: Injection. **(B)** Quantification of retrogradely labeled LOC neurons in the lateral, middle, and medial regions of the contralateral LSO in neonatally injected mice. N = 5 lateral region: 71 ± 30, middle region: 65 ± 31, medial region: 76 ± 28, one-way repeated measures ANOVA, F = 1.36, P = 0.3. **(C)** Comparison of the overall number of retrogradely labeled LOC neurons between the ipsilateral and the contralateral LSO in neonatally injected mice. N = 5 Paired t-test, t = 2.18, P = 0.09. **(D–F)** Region-specific comparisons between ipsilateral and contralateral LSO in neonatally injected mice. N = 5 Paired t-tests with Holm-Bonferroni correction for multiple comparisons. Lateral Region t = 1.1 P = 0.66. Middle region t = 1.01 P = 0.66. Medial region t = 2.16 P = 0.288. **(G)** Retrogradely labeled core LOC neurons in the contralateral LSO in juvenile mice injected with AAV2-retro. **(H)** Quantification of retrogradely labeled LOC neurons in the lateral, middle, and medial regions of the contralateral LSO in juvenile mice injected with AAV. N = 6 lateral region: 18 ± 9, middle region: 16 ± 12, medial region: 21 ± 15, one-way repeated measures ANOVA, F = 0.65, P = 0.54. **(I)** Comparison of the overall number of retrogradely labeled LOC neurons between the ipsilateral and the contralateral LSO in juvenile mice injected with AAV2-retro. N = 6 Paired t-test, t = 5.1, P = 0.0038. **(J–L)** Region-specific comparisons between ipsilateral and contralateral LSO in juvenile mice injected with AAV2-retro. N = 6 Paired t-test with Holm-Bonferroni correction for multiple comparisons. Lateral region t = 3.44 P = 0.018. Middle region t = 5.04 P = 0.01. Medial region t = 4.67 P = 0.01.

## 3 Results

### 3.1 Inner ear injections of AAV2-retro robustly transfect LOC neurons in neonatal and juvenile mice

Following neonatal semicircular canal injection of AAV2-retro (Figure 1A), we observed robust retrograde labeling of MOC neurons and extensive labeling of retrogradely transfected neurons in and around the ipsilateral LSO (Figure 1B). The large size and bi- or multipolar morphology of the neurons on the periphery of the LSO were consistent with a shell LOC identity (Warr et al., 1997; Darrow et al., 2006b; Wu et al., 2020), whereas the small, oval, and mostly fusiform morphology of the neurons positioned in the center of the LSO corresponded with the characterization of core LOC neurons in the literature (Campbell and Henson, 1988; Warr et al., 1997; Cantos et al., 2000; Brown and Levine, 2008; Vetter and Mugnaini, 1992). The number of retrogradely labeled core LOC neurons was 249 ± 115 (N = 5), which is in line with what has been reported previously for mice using retrograde tracing (112-307 neurons, Brown and Levine, 2008; Campbell and Henson, 1988) and with the number of core LOC neurons determined by immunohistochemistry using acetylcholinesterase as a marker (214 neurons, Brown and Levine, 2008). Our results thus indicate that a single injection of AAV2-retro into the PSCC of neonatal mice can label the majority of ipsilateral core LOC neurons.

The projections of core LOC neurons are tonotopically organized (Guinan et al., 1984), with neurons located in the lateral regions of the LSO projecting to the low-frequency apical regions of the cochlea and neurons located in the medial regions of the LSO projecting to the high-frequency, basal regions of the cochlea. Some AAV serotypes exhibit reduced transfection efficacy of cochlear regions distal from their injection site (György et al., 2017; Yoshimura et al., 2018; Zhang et al., 2023), while other serotypes exhibit varying levels of tropism for cells in the different regions of the cochlea (Iranfar et al., 2025; Ivanchenko et al., 2021). We quantified the number of retrogradely transfected LOC neurons in the corresponding lateral, middle, and medial regions of the LSO to determine whether or not AAV2-retro equally transfected LOC neurons along the entire length of the cochlea (Figure 1D). The medial region had the highest number of retrogradely labeled LOC neurons at 95 ± 43 followed by the lateral region at 79 ± 40 neurons and the middle region at 74 ± 36 (N = 5). The differences among the three regions, however, were not significant (one-way repeated measures ANOVA, F = 2.76, P = 0.12), which indicates that AAV2-retro is capable of spreading from the posterior semicircular canal to all cochlear regions and displays similar tropism to LOC neurons across cochlear regions.

The neuronal tropism of several AAV serotypes decreases with age of the host animal (Chakrabarty et al., 2013). In the mouse cochlea, the tropism of AAV-Anc80L65 and AAV-S for spiral ganglion neurons is higher in neonatal cochlea compared to later ages (Ivanchenko et al., 2021; Richardson et al., 2021). To investigate whether AAV2-retro still robustly transfects LOC neurons at older ages, we unilaterally delivered the viral vector to the inner ears of P21-28 mice. LOC neurons in the ipsilateral LSO of these mice were robustly transfected by AAV2-retro (Figure 1E), with an average of 178 ± 77 neurons (N = 6), which is within the range previously reported in the literature (112-307 neurons, Brown and Levine, 2008; Campbell and Henson, 1988) and is similar to the number we observed in neonatally injected mice (249 ± 115 N = 5; two-tailed t-test t = 1.407, P = 0.193). The number of retrogradely labeled LOC neurons in the lateral, middle, and medial regions of the ipsilateral LSO were similar at 57 ± 29, 59 ± 27, and 60 ± 29 respectively (Figure 1E, N = 6, one-way repeated measures ANOVA, F = 0.064 P = 0.937). These results indicate that AAV2-retro is an effective vector for retrograde transfection and gene delivery to core LOC neurons in both neonatal and juvenile mice.

### 3.2 Unilateral inner ear delivery of AAV2-retro results in transfection of LOC neurons in the contralateral LSO

Previous studies using a wide variety of anatomical tracers have demonstrated that the projections of core LOC neurons are almost entirely ipsilateral in mice (Campbell and Henson, 1988 horseradish peroxidase; Brown, 1993 biocytin and horseradish peroxidase; Brown and Levine, 2008 fluorogold and horseradish peroxidase). In contrast to these studies, our unilateral inner ear injections of AAV2-retro labeled core LOC neurons in the contralateral LSO in both neonatal (Figure 2A) and juvenile (Figure 2G) mice. Neonatal mice had an average of 212 ± 85 retrogradely labeled LOC neurons in the contralateral LSO (N = 5), which was similar to the 249 ± 115 neurons in the ipsilateral LSO (Figure 2C, paired t-test, t = 2.18, P = 0.09). Retrogradely transfected LOC neurons in the contralateral LSO were equally distributed along its mediolateral axis, with no significant differences observed between the lateral, middle, and medial regions (Figure 2B, one-way repeated measures ANOVA, F = 1.36, P = 0.3). Comparing the number of labeled LOC neurons in each of these LSO regions between the ipsilateral and contralateral LSO revealed no significant differences either (Figures 2D–F; paired t-test corrected for multiple comparisons; lateral P = 0.66; Middle P = 0.66; Medial P = 0.288).

In contrast to neonatal mice, in juvenile mice, the number of retrogradely labeled LOC neurons in the contralateral LSO (55 ± 31, N = 6) was significantly less than in the ipsilateral side (178 ± 77 neurons N = 6; Figures 2G–I, Paired t-test, t = 5.1, P = 0.0038). The reduction in the number of labeled neurons in the contralateral LSO was not confined to a single isofrequency region as the distribution of the labeled neurons was uniform across the mediolateral axis (Figure 2H; one-way ANOVA F = 0.65 P = 0.54) and all three individual LSO regions had significantly fewer LOC neurons labeled when compared to their ipsilateral counterparts (Figures 2J–L; paired t-test corrected for multiple comparisons; lateral P = 0.018; Middle P = 0.01; Medial P = 0.01).

### 3.3 AAV injected into the inner ear travels through the cerebrospinal fluid and into the contralateral inner ear

The extensive contralateral labeling of core LOC neurons following unilateral inner ear delivery of AAV2-retro was surprising in light of the ipsilateral nature of the LOC pathway reported earlier (Campbell and Henson, 1988; Brown, 1993; Brown and Levine, 2008). While contralateral labeling in juveniles was lower than in neonates, it remained substantial (∼23%) and far above the 0%–1% typically reported after unilateral tracer injections (Campbell and Henson, 1988; Brown, 1993; Brown and Levine, 2008). The perilymph of the cochlea is connected to the cerebrospinal fluid (CSF) of the central nervous system via the cochlear aqueduct (Kellerhals, 1979). This connection allows for the bidirectional flow of material (Talaei et al., 2019), including viral particles (Mathiesen et al., 2023), between the perilymph and the CNS. We hypothesized, therefore, that the robust labeling of LOC neurons in the contralateral LSO was most likely mediated by the diffusion of viral particles from the injected inner ear to the CSF via the cochlear aqueduct and onto the contralateral cochlea. To test our hypothesis, we surveyed the brains of mice that underwent inner ear AAV2-retro delivery for evidence of ectopic transfection of neurons that do not project to the cochlea and would have only been transfected by viral particles that diffused out of the cochlear perilymph into the CSF. Indeed, we observed ectopic transfection of cerebellar neurons in all the mice that underwent inner ear delivery of AAV2-retro as neonates (Figure 3A) or as juveniles (Figure 3B).

**FIGURE 3:**
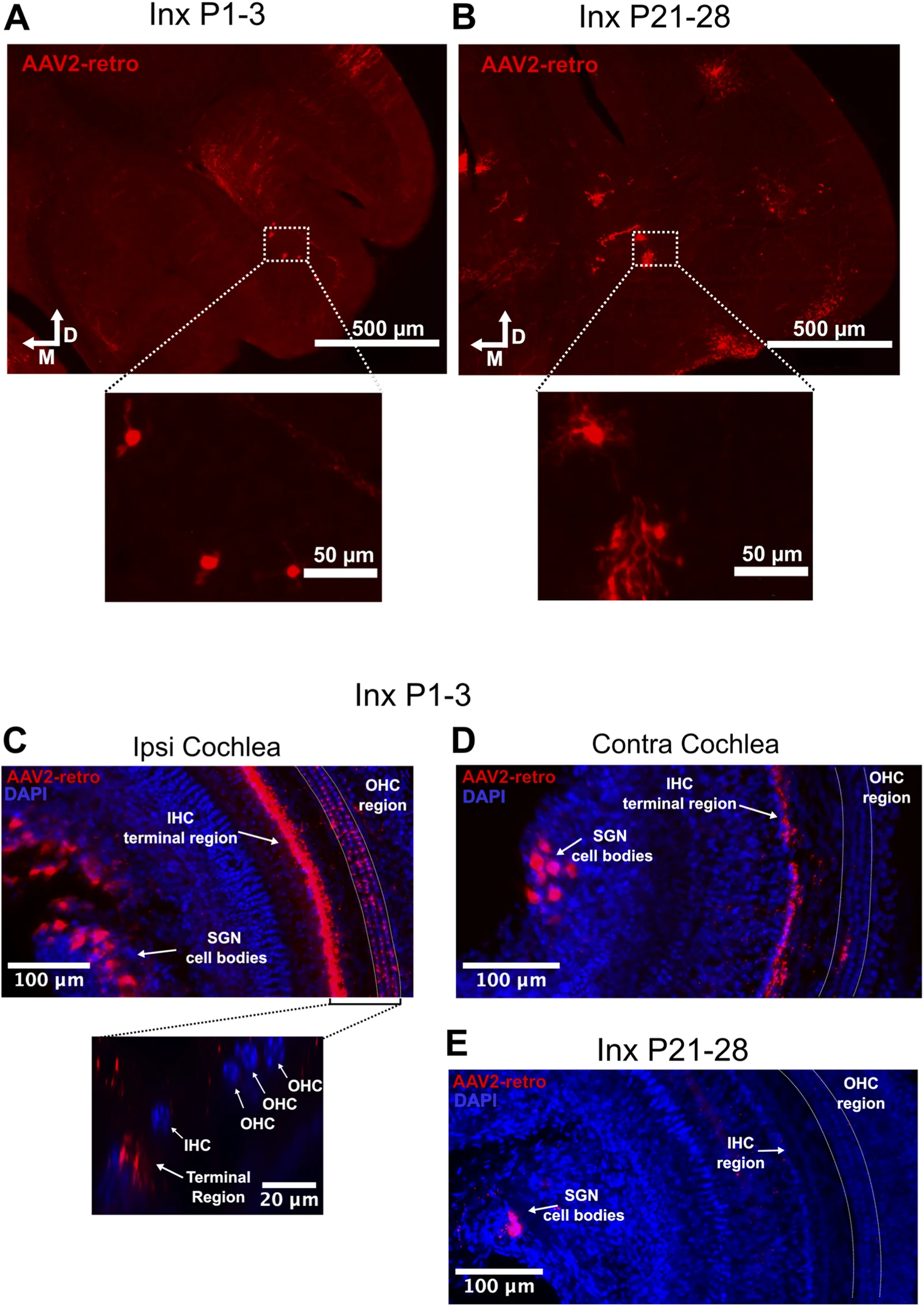
AAV2-retro injected into the inner ear diffuses throughout the cerebrospinal fluid and into the contralateral cochlea. **(A)** Transfected cerebellar neurons following neonatal inner ear delivery of AAV2-retro. M = medial, D = dorsal. **(B)** Transfected cerebellar neurons following juvenile inner ear delivery of AAV2-retro. **(C)** cochlea from the injected ear following neonatal inner ear delivery of AAV2-retro along with a magnified view of the organ of Corti cross-section showing AAV labeling of terminal synapses underneath the inner hair cell region. **(D)** Contralateral cochlea following neonatal inner ear delivery of AAV2-retro. **(E)** Contralateral cochlea following juvenile inner ear delivery of AAV2-retro.

To further test our hypothesis, we examined the injected cochlea for direct somatic transfection by AAV2-retro and whether we could observe similar transfection in the contralateral cochlea. In the injected cochlea, AAV2-retro robustly transfected the cell bodies of spiral ganglion neurons (SGN) (Figure 3C) and we observed transfected SGN cell bodies also in the contralateral cochlea in both neonatal animals (Figure 3D) and juveniles (Figure 3E). Taken together, these results suggest a diffusion of viral particles from the injected inner ear to the contralateral cochlea, where they infect the terminals of efferent fibers to retrogradely label LOC neurons in the contralateral LSO.

We next investigated whether lower doses of AAV2-retro can yield robust retrograde transfection of ipsilateral LOC neurons while minimizing the labeling of contralateral LOC neurons. To this end, we injected 200 nL and 50 nL of virus solution representing 50% and 12.5% of the original 360–400 nL dose. Reducing the dose of AAV2-retro still produced robust retrograde transfection of LOC neurons in the ipsilateral LSO (Figures 4A,C), with an average of 163 ± 74 (N = 5) and 181 ± 102 (N = 5) labeled LOC neurons for the 200nL and 50 nL respectively, which was similar to the 249 ± 115 neurons (N = 5) observed with the initial 400 nL dose (Figure 4E, one-way ANOVA F = 1.057 P = 0.378). However, lowering the dose of AAV2-retro significantly reduced the number of retrogradely labeled LOC neurons in the contralateral LSO (Figures 4B,D), with an average of 105 ± 32 (N = 5) and 63 ± 13 (N = 5) labeled neurons in the contralateral LSO for the 200nL and 50 nL respectively, compared to the 212 ± 85 neurons (N = 5) observed at the 400 nL dose (Figure 4F, one-way ANOVA, F = 10.28 P = 0.0025 Tukey *post hoc* comparison 200 VS 400 P = 0.02, 50 VS 400 P = 0.0023). Nevertheless, reducing the dose of AAV did not completely eliminate the diffusion of viral particles to the CSF as ectopic transfection of cerebellar neurons (Figure 4G) and contralateral SGNs (Figure 4H) was still observed even at the lowest 50 nL dose. Taken together, these results indicate that AAV2-retro maintains ipsilateral LOC transfection efficacy at lower doses and support the idea that contralateral labeling of LOC neurons is a dose-dependent consequence of viral particles diffusing through the CSF.

**FIGURE 4:**
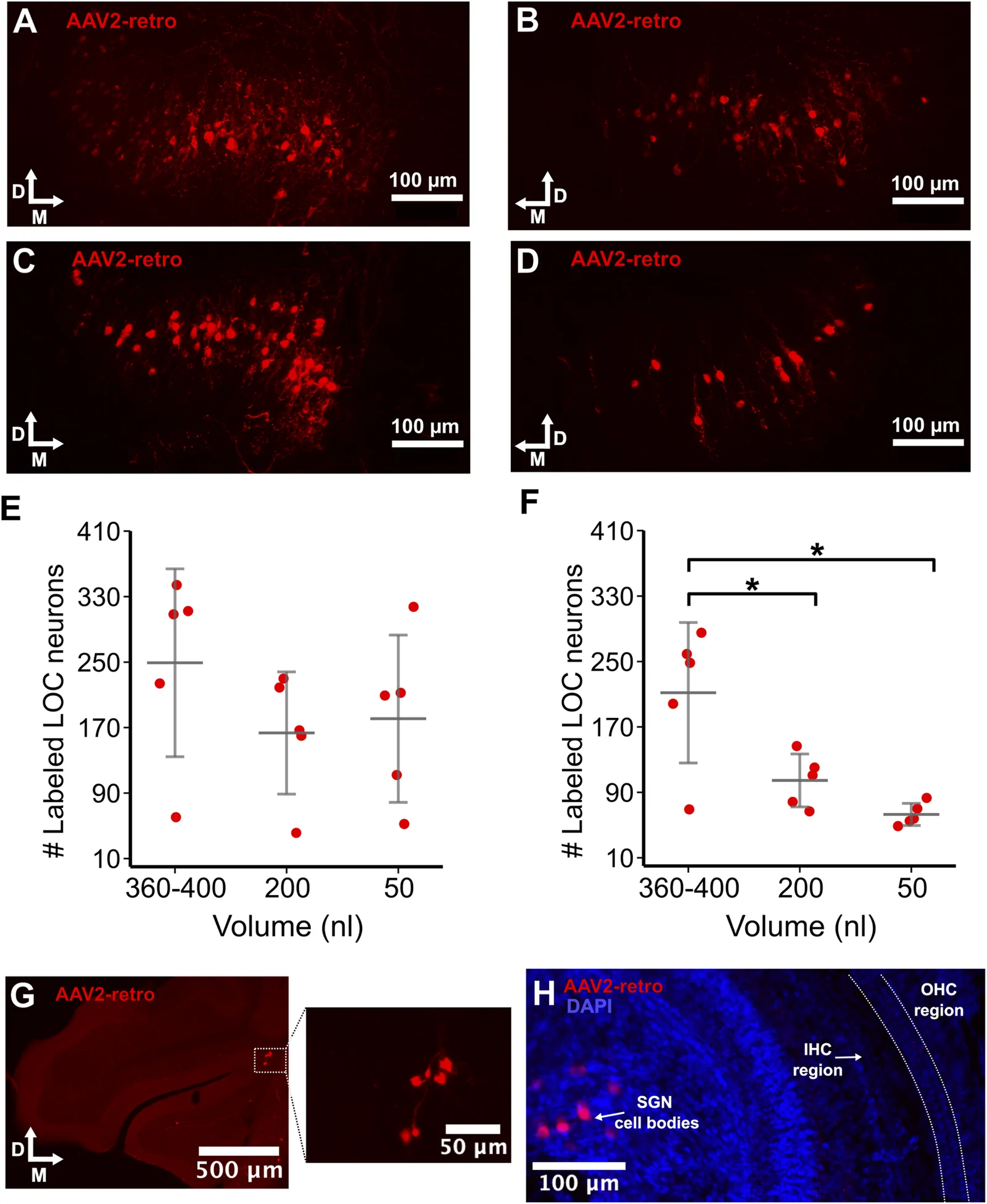
Reducing the dose of AAV2-retro still produced robust retrograde labeling of LOC neurons in the ipsilateral LSO but reduced labeling of LOC neurons in the contralateral LSO following neonatal unilateral inner ear delivery. **(A,B)** Retrogradely labeled core LOC neurons in ipsilateral and contralateral LSO, respectively, following neonatal inner ear delivery with 200 nL of AAV2-retro. M = medial, D = dorsal. **(C,D)** Retrogradely labeled core LOC neurons in ipsilateral and contralateral LSO, respectively, following neonatal inner ear delivery with 50 nL of AAV2-retro. **(E)** Quantification of retrogradely labeled core LOC neurons in ipsilateral LSO by each AAV volume injected. 360–400 nL: 249 ± 115 N = 5, 200 nL: 163 ± 74 N = 5, 50 nL: 181 ± 102 N = 5. One-way ANOVA F = 1.057 P = 0.378. **(F)** Quantification of retrogradely labeled core LOC neurons in contralateral LSO 360–400 nL: 212 ± 85 N = 5, 200 nL: 105 ± 32 N = 5, 50 nL: 63 ± 13 N = 5; One-way ANOVA, F = 10.28 P = 0.0025; Tukey *post hoc* comparison 200 nL VS 400 nL P= 0.02, 50 nL VS 400 nL P = 0.0023, 200 nL vs. 50 nL P = 0.46. **(G)** Transfected cerebellar neurons following neonatal inner ear delivery of 50 nL AAV2-retro. **(H)** Contralateral cochlea following unilateral inner ear delivery of 50 nL AAV2-retro.

### 3.4 Projections of core LOC neurons are exclusively ipsilateral in neonatal mice

In a variety of adult animal species 99%–100% of core LOC neurons project to the ipsilateral cochlea (Campbell and Henson, 1988; Brown, 1993; Brown and Levine, 2008). However, olivocochlear projections can undergo reorganization during early development (Bruce et al., 2000; Kaiser et al., 2011; Barone et al., 2019), which prompted us to investigate whether some core LOC neurons exhibit transient bilateral projections during early development, contributing to the contralateral labeling of LOC neurons we observed with AAV2-retro. To avoid transfection of the contralateral cochlea by AAV2-retro we used the chemical tracer Fast Blue, which has been previously used to trace olivocochlear efferents (Abou-Madi et al., 1987; Aschoff and Ostwald, 1987; Aschoff and Ostwald, 1988; Aschoff et al., 1988; Azeredo et al., 1999; Cantos et al., 2000; Horvath et al., 2000) and is not susceptible to off-target labeling in the contralateral cochlea. Unilateral injection of Fast Blue into the cochlea of neonatal mice resulted in 157.8 ± 38.7 (N = 4) retrogradely labeled core LOC neurons in the ipsilateral LSO, which was comparable to the number we observed in mice that received 50 nL AAV2-retro (181 ± 102) (Figures 5A,C; t-test, t = 0.46 P = 0.66). However, Fast Blue labeled very few core LOC neurons in the contralateral LSO (Figure 5B) with an average of 3 ± 1.8 (N = 4) neurons, which was substantially less than the 63 ± 13 neurons (N = 5) observed with the 50 nL AAV2-retro dose (Figure 5D; t-test, t = 9.79 P = 0.0005). The sparse contralateral LSO labeling is not due to a failure of the tracer to label long-distance neurons because MOC neurons in the contralateral SOC were robustly labeled by Fast Blue (Figure 5B). Indeed, the number of retrogradely labeled MOC neurons in the contralateral SOC was larger than that of MOC neurons in the ipsilateral SOC at 24 ± 7.6 and 10 ± 3.9 respectively (Figure 5E; N = 4, paired t-test, P = 0.0035 t = 6.653), which is in line with past literature reports demonstrating that the MOC pathway in mice is 63%-75% contralateral (Campbell and Henson, 1988; Maison et al., 2003; Brown and Levine, 2008).

**FIGURE 5:**
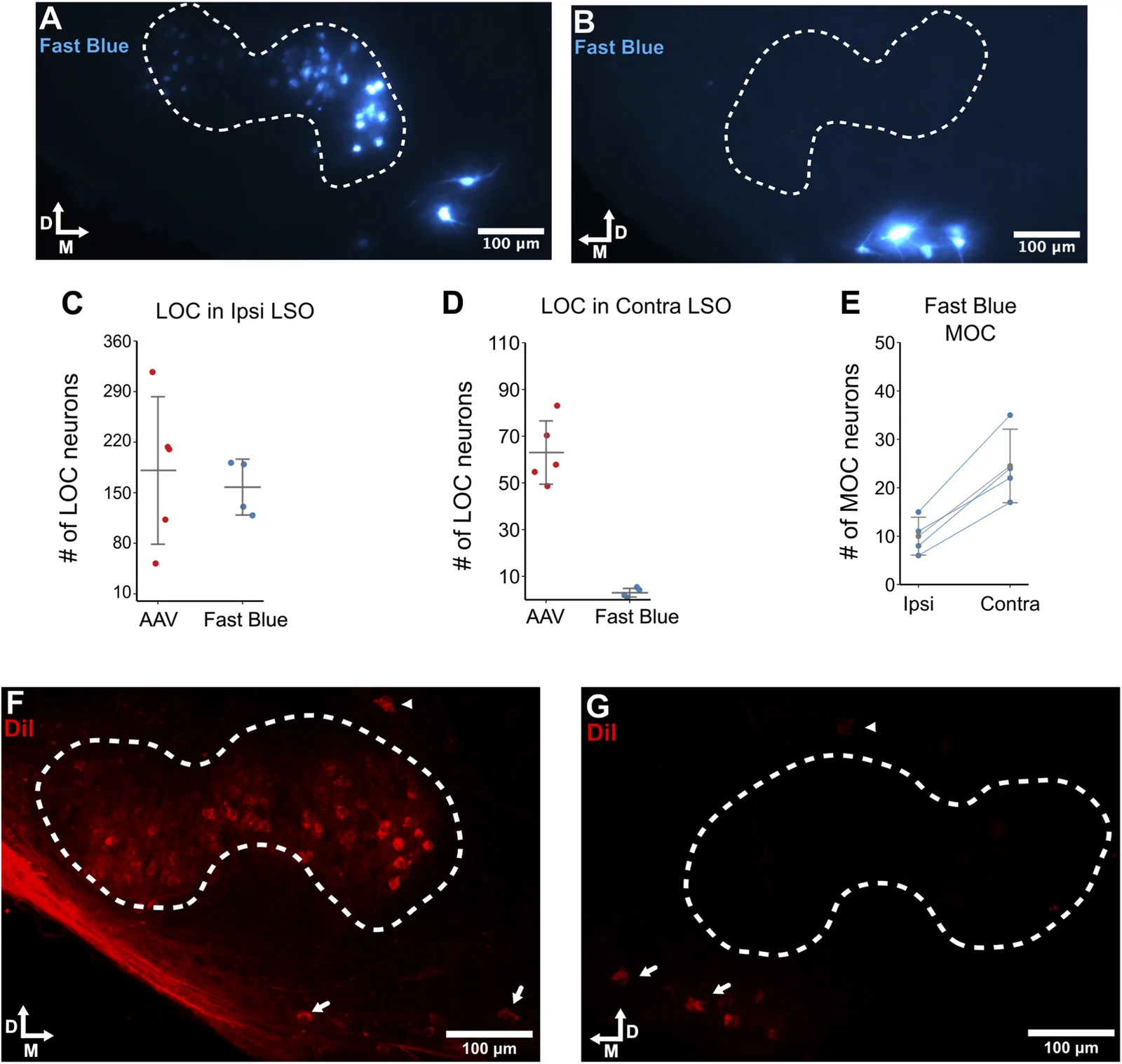
Neonatal unilateral inner ear injections of Fast Blue and DiI result in ipsilateral labeling of core LOC neurons and bilateral labeling of MOC neurons. **(A,B)** Retrogradely labeled OC neurons in the ipsilateral and contralateral SOC, respectively, following unilateral inner ear injection of Fast Blue. M = medial, D = dorsal. **(C)** Comparison of the number of retrogradely labeled LOC neurons in the ipsilateral LSO following 50 nL AAV2-retro and Fast Blue delivery to the inner ear. 50nL AAV2-retro: 181 ± 102 (N = 5); Fast Blue: 157.8 ± 38.7 (N = 4); t-test, t = 0.46 P = 0.66. **(D)** Comparison of the number of retrogradely labeled LOC neurons in the contralateral LSO following 50 nL AAV2-retro and Fast Blue delivery to the inner ear 50 nL AAV2-retro: 63 ± 13 (N = 5); Fast Blue: 3 ± 1.8 (N = 4); t-test, t = 9.79 P = 0.0005. **(E)** Quantification of retrogradely labeled MOC neurons following Fast Blue delivery to the inner ear. Ipsilateral SOC: 24 ± 7.6; Contralateral SOC: 10 ± 3.9; (N = 4) paired t-test, P = 0.0035 t = 6.653. **(F,G)** Retrogradely labeled OC neurons in the ipsilateral and contralateral SOC, respectively, following unilateral DiI infusion of the inner ear. Arrowheads: Shell LOC neurons. Arrows: MOC neurons.

Fast Blue tracing requires endosomal uptake by intact axon terminals (Köbbert et al., 2000) and active axonal retrograde transport (Johnston et al., 1986), which may favor the labeling of thick MOC axons over the thinner LOC axons leading to more labeling of MOC neurons in the contralateral SOC compared to LOC. We thus conducted a final set of retrograde tracing experiments using DiI, a lipophilic tracer that diffuses passively in the lipid bilayer layer of neurons and does not depend on endosomal uptake or active axonal transport. DiI has been previously used to reliably trace the afferent and efferent innervation of the cochlea in neonatal animals (Kandler and Friauf, 1993; Bruce et al., 2000). In the LSO ipsilateral to the DiI-infused cochlea, core LOC neurons were retrogradely labeled with DiI throughout the entire length of the LSO (Figure 5F), whereas the contralateral LSO contained little to no retrogradely labeled core LOC neurons (Figure 5G). As was the case with the Fast Blue tracing experiment, the labeling of MOC neurons was robust both in the ipsilateral (Figure 5F) and contralateral (Figure 5G) SOC. Taken together, the results obtained with DiI and Fast Blue tracing indicate that the efferent projections of core LOC neurons are indeed ipsilateral in neonatal mice and that most of the contralateral LOC labeling we observed following unilateral delivery of the smallest, 50nL, dose tested can be reliably attributed to the diffusion of viral particles from the injected ear.

## 4 Discussion

In this study, we investigated the efficacy of the AAV2-retro serotype in retrogradely transfecting core neurons of the LOC pathway. We report that injection of AAV2-retro into the posterior semicircular canal in neonatal and juvenile mice produced robust transfection of olivocochlear efferent neurons in the SOC, including core LOC neurons along the entire mediolateral axis of the LSO (Figures 1B,D), which demonstrates that AAV2-retro can transfect the terminals of projecting LOC axons across all turns of the cochlea. Transgene expression has been reported to persist for at least 1 year following AAV2 (Gullapalli et al., 2026) and AAV9 (Bailey et al., 2018) neuronal transfection. While we did not specifically set out to investigate the persistence of transgene expression in this study, we did observe that transgene expression was still detectable for up to 4 months in LOC neurons transduced with AAV2-retro-hM3D(Gq)-mCherry (unpublished data). The results reported in this study differ from those of Wang et al. (2021), who did not report transfected LOC neurons after AAV2-retro injections into the inner ear. The most likely reason for this apparent discrepancy is that Wang et al. (2021) were primarily concerned with the role of MOC and periolivary efferents, which, in the mouse, are distributed along a much wider rostrocaudal extent compared to core LOC neurons and the LSO (Brown and Levine, 2008). It is thus possible that the brainstem sections where Wang et al. (2021) confirmed the presence of robust viral expression in MOC and periolivary neurons did not contain the LSO nucleus.

Unexpectedly, we also found that unilateral inner ear injection of AAV2-retro produced persistent labeling of contralateral LOC neurons (Figures 2–4). Our tracing with Fast Blue and DiI indicates that bilateral labeling of the LOC pathway was not a result of hitherto unknown bilateral projections of LOC neurons or that it indicated a transient developmentally regulated contralateral projection, but that it most likely stemmed from the ability of AAV2-retro to diffuse through the cochlear aqueduct to the CNS and from there into the contralateral cochlea. This conclusion is supported by the fact that the cochlear aqueduct connects the CSF of the CNS to the perilymph of the cochlea (Kellerhals, 1979; Gopen et al., 1997; Talaei et al., 2019). We also cannot exclude that some viral particles may have spread beyond the CNS, as AAV delivery to the CSF has been reported to result in widespread biodistribution beyond the CNS, with AAV particles detected in heart, lung, liver, and spleen following intracisternal (Hordeaux et al., 2018), lumbar (Gray et al., 2013; Hinderer et al., 2014) and inner ear injections of AAV (Andres-Mateos et al., 2022; Ishibashi et al., 2025). Whether the AAV2-retro used in our study also spread to internal body organs remains a question for further studies.

We report that the extent of contralateral cochlear transfection, likely achieved via systemic viral spread throughout the CSF, was directly correlated to the amount of AAV2-retro delivered to the inner ear (Figure 4F). This observation is consistent with prior literature reports on varying levels of transfection in the contralateral cochlea following unilateral delivery of AAV. For example, György et al. (2018) reported contralateral transfection in only 20% of the animals following delivery of 1-1.2 ul of AAV-PHP.B at a titer of 5-18e+10 GC/mL, whereas studies that delivered similar volumes of AAV-PHP.B but at higher titers (1.07e+12 to 1.10e+13 GC/mL) reported observing contralateral transfection in all the animals that underwent unilateral inner ear delivery of the virus (Keppeler et al., 2018; Bali et al., 2021; Mittring et al., 2023). We also report that age plays an important role in determining the extent of systemic CNS spread and contralateral cochlear transfection as unilateral inner ear delivery of AAV in neonatal mice resulted in a much more robust transfection of LOC efferents in the contralateral cochlea (Figures 2C–F) compared to what was observed following inner ear delivery in juvenile mice (Figures 2I–L). Our observation that AAV achieves much higher systemic spread in the neonatal CNS is in line with previous studies showing that delivery of AAV to the CSF in neonates results in much more widespread and robust transfection in the CNS compared to adults (von Jonquieres et al., 2013; Mathiesen et al., 2020; Garza et al., 2025).

The most important factor behind systemic viral spread to the CNS and the contralateral cochlea, however, is arguably the AAV serotype utilized. When the same titer and volume of AAV are delivered to the CSF in animals of the same age, some AAV serotypes achieve much higher rates of widespread and robust transfection of CNS neurons compared to others (Chakrabarty et al., 2013; Hammond et al., 2017; Chauhan et al., 2024). Indeed, AAV serotypes that have been reported to achieve transfection of the contralateral cochlea following unilateral inner ear delivery like AAV-Anc80L65 (Landegger et al., 2017) and AAV-PHP.B (Keppeler et al., 2018; Bali et al., 2021; Mittring et al., 2023), have also been reported to achieve more widespread transfection of CNS neurons following delivery to the CSF compared to other serotypes (Deverman et al., 2016; Hudry et al., 2018; Mathiesen et al., 2020). The AAV2-retro serotype was created from AAV2 by inserting a sequence of 10 amino acids and introducing two point mutations in the VP1 capsid gene (Tervo et al., 2016). Compared to other AAV serotypes that transduce neuronal tissue, AAV2 exhibits some of the lowest levels of systemic spread throughout the CNS following delivery to the inner ear (Han et al., 2024), CSF (Passini et al., 2003; Broekman et al., 2006), and bloodstream (Zincarelli et al., 2008). The modification of the AAV2 capsid gene, therefore, not only enhanced the retrograde transfection efficacy of AAV2-retro, but also increased its propensity for systemic spread throughout the CNS.

AAV2-retro exhibits an attenuated binding affinity for heparan sulfate proteoglycans (HSPG) compared to its ancestral serotype, AAV2 (Tervo et al., 2016). HSPGs can be broadly classified into transmembrane HSPGs, like syndecan and glypican, that are found on the cell surface and secreted HSPGs, like perlecan and agrin, that are present in the extracellular matrix (ECM) (Condomitti and de Wit, 2018; Kamimura and Maeda, 2021; Matsuzaka and Yashiro, 2024). The strong HSPG binding affinity of AAV2 (Summerford and Samulski, 1998; Opie et al., 2003; Perabo et al., 2006; Lochrie et al., 2006; O’Donnell et al., 2009) leads to its sequestration by ECM-bound HSPGs, which in turn limits its ability to spread from its injection site (Cehajic-Kapetanovic et al., 2011). Accordingly, attenuation of the HSPG binding affinity enhances the spread of AAV2 from its injection site (Gorbatyuk et al., 2019). The high expression of ECM-bound HSPGs in cochlear basement membrane tissue (Torihara et al., 1994; Cosgrove et al., 1996; Tsuprun and Santi, 2001) coupled with the high affinity that AAV2 has for HSPG could explain why, compared to other serotypes, AAV2 produces some of the lowest levels of systemic spread throughout the CNS following unilateral inner ear delivery (Han et al., 2024). Conversely, the attenuated HSPG binding affinity of AAV2-retro likely prevented its sequestration to the ECM of the injected cochlea’s tissue and contributed to its ability to transfect neurons throughout the CNS and to robustly transfect SGN cell bodies and LOC efferent terminals in the contralateral cochlea (Figures 2–4).

In addition to the difference in their propensity for systemic spread throughout the CNS, AAV2-retro and its ancestral serotype, AAV2, also seem to differ when it comes to their cellular tropism in the cochlea. Previous studies have shown that delivery of AAV2 to the inner ear results in the transfection of inner and outer hair cells (Kilpatrick et al., 2011; Iranfar et al., 2025). However, we report that AAV2-retro failed to transfect either hair cell type, as it primarily transfected efferent terminals and SGN cell bodies in the cochlea (Figure 3D). Even though AAV2 achieves cellular entry through interacting with the receptor AAVR (Pillay et al., 2016; Meyer et al., 2019), the HSPG binding affinity of AAV2 can influence its tropism through determining the extent of its sequestration on the surface of cells with rich expression of HSPGs (Perabo et al., 2006). Indeed, attenuating the HSPG binding affinity of AAV2 reduces its transfection efficacy of these cells (Summerford and Samulski, 1998; Opie et al., 2003; Perabo et al., 2006; Lochrie et al., 2006). HSPG expression in cochlear hair cells plays an important role in development (Freeman et al., 2015) and has been proposed to underlie the differences in viral transfection efficacy between outer and inner hair cells (Iranfar et al., 2025). It is possible, therefore, that the attenuated HSPG binding affinity of AAV2-retro contributed to its reduced cellular tropism for hair cells. It is worth noting, however, that AAV serotypes which exhibit weak HSPG binding affinity, like AAV8 (Opie et al., 2003; Wu et al., 2006), can also successfully transfect hair cells (Kilpatrick et al., 2011). As such, while the attenuated HSPG binding affinity of AAV2-retro may have contributed to its inability to transfect hair cells, it is very unlikely that HSPG binding affinity was the only factor behind the change in the cellular tropism of AAV2-retro.

Taken together, our results indicate that the AAV2-retro is an effective serotype for retrograde gene delivery to core LOC neurons. We report, however, that AAV2-retro differs from its ancestral serotype, AAV2, in other properties beyond its enhanced capacity for retrograde transfection. Most consequential of these differences was that the inner ear delivery of AAV2-retro resulted in a level of systemic spread throughout the CNS and contralateral cochlea that far exceeded what has been reported for AAV2 (Han et al., 2024). The propensity of AAV2-retro to spread throughout the CSF following inner ear delivery necessitates future investigation of potential neuroinflammatory consequences as delivery of AAV to the CSF was reported to result in immune cell infiltration and neurodegeneration in the dorsal root (Tukov et al., 2022) and trigeminal (Hordeaux et al., 2018) ganglia as well as an increase in serum neutralizing antibodies against AAVs (Hordeaux et al., 2018). Finally, insufficient consideration of the ability of AAV2-retro to spread far from its injection site could, therefore, result in erroneous interpretation of results. For example, AAV2-retro delivery to the hippocampus was reported by Bian et al. (2019) to label anterior cingulate area (ACA) efferent projections to the hippocampus; however, follow-up studies that utilized AAV2 (Glat et al., 2022) and AAV2/9 (Shi et al., 2022) as anterograde tracers did not observe these ACA to hippocampus projections. Finally, Andrianova et al. (2023) demonstrated through the use of Fast Blue and meticulous analysis of AAV2-retro transfection pattern that the previous report of ACA efferent projections to the hippocampus (Bian et al., 2019) was almost certainly caused by off-target AAV2-retro transfection. In conclusion, it is imperative to carefully consider the propensity of AAV2-retro to spread far from its injection site when planning experiments in order to utilize a titer and volume that minimizes the risk of off-target labeling. Additionally, our study contributes to a growing body of literature highlighting the need to carefully verify any unexpected projections discovered using AAV2-retro, and other even more virulent retrograde AAV serotypes (Lin et al., 2020; Han et al., 2023), through the use of a second, preferably chemical, tracing approach.

## Data Availability

The raw data supporting the conclusions of this article will be made available by the authors, without undue reservation.
